# Association of XRCC3, XRCC4, BAX, and BCL-2 Polymorphisms with the Risk of Breast Cancer

**DOI:** 10.1155/2022/5817841

**Published:** 2022-03-14

**Authors:** Emre Ozoran, Fadime Didem Can Trabulus, Duygu Erhan, Bahadir Batar, Mehmet Guven

**Affiliations:** ^1^Department of General Surgery, School of Medicine, Koc University, Istanbul, Turkey; ^2^Department of General Surgery, Bahcesehir University Faculty of Medicine, Istanbul, Turkey; ^3^Department of Medical Biology, Cerrahpasa School of Medicine, Istanbul University Cerrahpasa, Istanbul, Turkey; ^4^Department of Medical Biology, Tekirdag Namik Kemal University School of Medicine, Tekirdag, Turkey

## Abstract

**Background:**

Breast cancer is the most common malignancy in women. Genetic risk factors associated with breast cancer incidence have been identified.

**Aims:**

This study is aimed at determining the association of XRCC3 Thr241Met (rs861539), XRCC4 G(-1394) T (rs6869366) DNA repair and BAX G(-248) A (rs4645878), and BCL2 C(-938) A (rs2279115) apoptotic gene polymorphisms with breast cancer.

**Materials and Methods:**

Genetic analysis was performed using peripheral blood samples. Gene polymorphisms were detected by using polymerase chain reaction-restriction fragment length polymorphism (PCR-RFLP) technique. 175 patients and 158 healthy controls were enrolled in the study.

**Results:**

Breast cancer risk was 5.43 times more in individuals with AA genotype of Bax G(-248) A (rs4645878) (*P* = 0.002). The risk of metastasis was 11 times with this genotype. It was associated with 6 times more risk of having a tumor larger than 2 cm. The risk of breast cancer was 2.77 times more in individuals carrying the Met/Met genotype of XRCC3 Thr241Met (rs861539) (*P* = 0.009). The risk of having advanced clinical stage (stage III+IV) with the Met/Met genotype was 4 times more increased. No relationship with breast cancer was found with XRCC4 G(-1394) T (rs6869366) and BCL2 C(-938) A (rs2279115) gene polymorphisms.

**Conclusion:**

Multicenter trials using subjects with genetic variations are needed to establish the relationship between breast cancer and single gene polymorphism.

## 1. Introduction

Breast cancer is one of the most common malignancies seen in women and one of the leading reasons of cancer-related mortality in developed countries. The lack of clear knowledge about the molecular mechanisms responsible for the development of breast cancer empowers the need for detailed and all round studies on this subject. Although its etiology is not clearly known, several genetic risk factors related to the high incidence have been defined. Studies have suggested that DNA repair and apoptosis mechanisms could have a role in the development of breast cancer. It has been reported that DNA repair and apoptosis gene polymorphisms could affect breast cancer risk [1, 2].

DNA repair mechanisms play major roles in the sustainability of genomic integrity. Various types of DNA damages have been repaired with various types of DNA repair mechanisms. DNA double-strand breaks could result from factors like free radicals of endogenous origin, exogenous chemicals, and ionizing radiation [3]. Mammal cells have established two different pathways for the repair of DNA double-strand breaks, homologous recombination (HR) and non-homologous end joining (NHEJ). Epidemiological studies have shown that DNA double-strand breaks are a risk factor in the development of breast cancer [4]. These findings, put the genes responsible for DNA double-strand break repair important candidates for further studies.

Aiding in preserving the stability of the chromosome, X-ray repair cross-complementing group 3 (XRCC3) gene is enrolled the HR pathway [5]. The product protein is enrolled in preserving the stability of the chromosome and in case of DSBs mending the DNA damage. XRCC3 gene has been mapped at 14q32.3 of the human chromosome. XRCC3 protein interacts with Rad51 during the repair process of DNA double-strand breaks aiding in the sustainability of DNA [6]. The X-ray cross-complementing group 4 (XRCC4) is an important component of NHEJ. XRCC4 gene has been mapped at 5q13-q14 of the human chromosome. XRCC4 protein forms a complex interacting with DNA ligase IV in the repair process of DNA double-strand breaks [7]. This complex is responsible for the ligation step of NHEJ repair. Single-nucleotide polymorphisms (SNPs) occurring in the XRCC3 and XRCC4 genes could enhance the injury caused by the unrepaired DNA damage leading to inclination to malignancy.

Apoptosis is programmed cell death at physiologic and pathologic circumstances. The disruptions in the apoptotic pathways could lead to development of cancer by affecting cellular hemostasis [8]. Apoptotic process is regulated by several proapoptotic or antiapoptotic proteins. Bcl-2 is a proapoptotic protein while Bax is an antiapoptotic protein. The levels of these two proteins are important indicators in the rate of apoptosis. BCL-2 gene has been mapped at 18q21.3 in the human chromosome. BCL-2 C(-938) A polymorphism at the promoter region of BCL-2 gene is the most common polymorphism. This polymorphism has been associated with predisposition to breast cancer [6, 9]. BAX gene has been mapped at 19q13.3. BAX G(-248) A polymorphism at the promoter region of the Bax gene has been associated with decreased Bax expression [10]. Many studies showed that BAX G(-248) A polymorphism has been associated with the risk of several cancers [11–13].

In our study, we investigated the relationship between breast cancer risk and genetic variations in DNA repair [XRCC3 Thr241Met and XRCC4 G(‐1394) T] and apoptosis [BAX G(-248) A and BCL-2 C(‐938) A] pathways. Our results could aid in linking the presence of gene polymorphism with clinical findings.

## 2. Methods

### 2.1. Study Population

The study population consisted of 175 female patients with breast cancer who admitted to the breast disease outpatient clinic of Istanbul Education and Research Hospital. The control group consisted of 158 women with the same demographic status as the disease group. The demographical information of the patients was obtained by one on one interviews. Histopathological diagnosis and data were obtained with the permission of the Pathology Department. The distributions of clinical characteristics of the patients are shown in Table 1.

The study was approved by the ethics committee of Istanbul Education and Research Hospital. The funding for genetic analysis was provided by the Education and Planning Committee of the same hospital. Genetic analysis was performed in Department of Medical Biology, Cerrahpasa School of Medicine, Istanbul University-Cerrahpasa.

### 2.2. Extraction of DNA and Genotyping Analysis

Blood samples were taken into vacuumed, sterile K3-EDTA tubes (2 ml), and stored at −20°C until analysis. At the day of analysis, total genomic DNAs were prepared using DNA isolation kit (High Pure PZR Preparation Template kit, Roche Diagnostics GmbH, Mannheim, GE) according to the manufacturer's instructions.

Genotyping of XRCC3 Thr241Met [14], XRCC4 G(-1394) T [15], BAX G(-248) A [16], and BCL2 C(-938) A [17] was determined by using the polymerase chain reaction-restriction fragment length polymorphism (PCR-RFLP) assay. PCR was initially performed to determine the polymorphic regions using suitable primers. Each PCR was performed in a total volume of 25 *μ*l reaction mixture containing 100 ng DNA, 1 X PCR buffer (with KCI), 0.04 mM dNTPs, 0.04 U Taq DNA polymerase, 10 pmol forward and reverse primers, and variable amount of H_2_O. The PCR conditions were presented in Table 2. PCR products were further subjected to digestion with restriction enzymes (Table 3). The PCR products were visualized by electrophoresis through a 3% agarose gel. The relative size of the PCR products was determined through comparison of the migration of a 50–1000 bp DNA molecular weight ladder (Invitrogen, Grand Island, NY, USA). In the event of any conflicts, the genotypes were repeated.

### 2.3. Statistical Analysis

Mean and standard deviations (SDs) were shown as continuous variables. Student's *t*-test was used in portraying the differences among two continuous variables. Chi square (*χ*^2^) or Fischer's exact test (two sided) were utilized in evaluation the genotypes and alleles, and test for deviation of genotype distribution from Hardy–Weinberg equilibrium (HWE). *P* values of <0.05 were considered statistically significant. The odds ratio (OR) and their 95% confidence intervals (CIs) were calculated to estimate the strength of the association. The data were analyzed using Statistical Package for the Social Sciences (SPSS; version 18.0).

## 3. Results

Our study consisted of 175 patients and 158 healthy controls. Patients (53 ± 12 years, range from 21 to 84 years) and controls (54 ± 9 years, range from 25 to 85 years) were not different in terms of age (*P* = 0.44). Smoking rate was 35% in the patients and 37% in the controls. Smoking status was not significantly different between patients and controls (*P* = 0.81).

The distributions of the XRCC3 Thr241Met, XRCC4 G(-1394) T, BAX G(-248) A, and BCL2 C(-938) A genotypes were in accordance with the HWE among the cases and controls.

In the analysis of BAX G(-248) A gene polymorphism, homozygote expression (AA genotype) of BAX-248A allele was associated with 5 times increased risk of breast cancer (OR = 5.43, 95% CI = 1.70–15.84; *P* = 0.002). BAX-248 AA genotype was seen in 3% of the control group and 14% of the patients. The frequency of the G allele was 69% in the patients and 74% in the controls. The frequency of the A allele was 31% in the patients and 36% in the controls. The difference was not statistically significant (Table 4). In the analysis of XRCC3 Thr241Met polymorphism, women with homozygote expression (Met/Met genotype) of 241Met allele had three times increased risk of breast cancer (OR = 2.77, 95% CI = 1.26–6.11; *P* = 0.009). XRCC3 241 Met/Met genotype was seen in 9% of the controls and 19% of the patients. The frequency of the XRCC3 241Thr allele was 55% in the patients and 65% in the controls. The frequency of the XRCC3 241Met allele was 45% in the patients and 35% in the controls. These results were not statistically significant (Table 4). No such significant difference between groups was observed for neither the genotypes nor the alleles of BCL2 C(-938) A and XRCC4 G(-1394) T polymorphisms (Table 4).

We investigated the association between the clinical characteristics of the patients and XRCC3 Thr241Met, XRCC4 G(-1394) T, BAX G(-248) A, and BCL2 C(-938) A genotypes. The BAX G(-248) AA genotype defined as the risk genotype was associated with metastatic status (*P* = 0.02) and tumor size (*P* = 0.02). Patients with AA genotype had 11 times increased risk of having metastasis (OR: 10.8, 95% CI:1.40–82.7). The patients with the AA genotype had 6 times increased risk of having tumor sizes more than 2 cm (OR: 6.1, 95% CI:1.2–30.0). In addition, XRCC3 241 Met/Met genotype defined as the risk genotype was associated with clinical stage (*P* = 0.02). Patients with Met/Met genotype had 4 times increased risk of being clinical stage III + IV (OR: 3.85, 95% CI:1.20–12.7). On the other hand, XRCC4 G(-1394) T and BCL2 C(-938) gene polymorphism and all of the disease parameters did not have any statistically significant relationship. Also, we did not find any significant relationship between XRCC3 Thr241Met, XRCC4 G(-1394) T, BAX G(-248) A, and BCL2 C(-938) A polymorphisms and the ER/PR/HER2 and triple negative status of the patients (*P* > 0.05, data not shown).

## 4. Discussion

We investigated the relationship between the risk of breast cancer and XRCC3 Thr241Met, XRCC4 G(-1394) T, BAX G(-248) A, and BCL2 C(-938) A gene polymorphisms. These genes code for the proteins enrolled in DNA injury repair and apoptosis which are important processes in carcinogenesis. Several studies addressed the polymorphisms on these genes. The results from the previous studies show variations. The genetic differences endemic in a geographical area could be one of the reasons. The frequency of genetic variants associated with a gene polymorphism in a particular population is an important determinant of the breast cancer risk. Thus, differences in the incidence of variant alleles associated with a polymorphism among societies may lead to different results. The genes we studied encode proteins registered in DNA damage repair and apoptosis, which are important processes in carcinogenesis and breast cancer. The polymorphisms we have studied regarding these genes are polymorphisms that have been found to be related to different parameters and cancer risk in various cancers but have not been studied in the Turkish population.

In our study, women with homozygote BAX-248A allele (AA genotype) had 5 times more risk of developing breast cancer. In addition, status of metastasis and tumor size was associated with this genotype. These findings were similar with the study of Kholoussi et al. [18]. They found that presence of heterozygote variant BAX-248A allele (GA genotype) was associated with higher grade (grade 3 or more), T2 status and having lobular disease. Similar results were obtained in homozygote and heterozygote BAX-248A variant alleles (GA genotype+AA genotype), thus making BAX-248A variant allele as the “risk allele”.

The relationship between BAX G(-248) A gene polymorphism and clinical parameters in different types of cancer has been studied previously with various different results. Wang et al. studied the effects of BAX G(-248) A gene polymorphism and survival in gastric cancer patients receiving postoperative chemotherapy. In their study, having at least one variant genotype in BAX G(-248) A was associated with increase in the recurrence risk and poorly affecting survival [19]. Gu et al. studied the relationship of BAX G(-248) A gene polymorphism and hematological toxicity in patients with advanced stage small cell lung cancer receiving platinum based chemotherapy. They showed that BAX G(-248) A gene polymorphism did not affect survival [20].

The analysis we conducted on XRCC3 Thr241Met polymorphism portrayed that, homozygote expression of 241Met allele (241Met/Met) was associated with 3 times increased risk of developing breast cancer. This genotype was also associated with clinical stages of III + IV. Chai et al. performed a meta-analysis on XRCC3 Thr241Met gene polymorphism and breast cancer arriving at similar results as our study. In that study having 241 Met/Met genotype in XRCC3 Thr241Met gene polymorphism was reported as a risk factor for breast cancer especially in the Asian population [11, 21]. Qureshi et al. studied the effects of XRCC3 (Thr241Met) gene polymorphism and breast cancer, and their results were similar with our study [22]. In their study 241Met/Met genotype was associated with 1.5 times increased risk of developing breast cancer. Similar results as our study were obtained by Jara et al., who studied the effects of XRCC3 Thr241Met gene polymorphism and breast cancer [23]. It was shown that XRCC3 241Met allele carriers had increased risk of developing breast cancer. Smith et al.'s study on the same relationship with breast cancer patients and healthy controls was in parallel with our study, finding to relationship with XRCC3 Thr241Met gene polymorphism and breast cancer [24]. On the other hand, in the study by Romanowicz et al. investigating the relationship between DNA repair gene polymorphisms and breast cancer; XRCC3 Thr241Met gene polymorphism was not associated with the risk of breast cancer [25]. The relationship between XRCC3 Thr241Met gene polymorphism and breast cancer was not studied previously. Nonetheless, Ji et al. showed that XRCC3 Thr241Met gene polymorphism did not have an effect on response to chemotherapy treatment and overall survival in osteosarcoma patients [26].

In our study, we did not find a relationship with BCL2 C(-938) A gene polymorphism and breast cancer like Searle et al. [27]. Neither breast cancer nor the lymph node involvement related survival was associated with this polymorphism. Zhang et al. conducted a study on investigating the relationship between BCL2 C(-938) A gene polymorphism and breast cancer. They found that patients with AA genotype had 2.37 times more risk of developing breast cancer than people with AC and CC genotypes [28]. This genotype was related with lymph node positivity and pathological diagnosis. The incidence of the genotypes in the study group (based on the control group) was 7.5% was AA, 49.5% was AC, and 43% was CC. In our study, 31% was AA, 48% was CA, and 21% was AA genotype in the control group. The difference in the incidence of AA genotype between the two populations could be the reason for the absence of a relationship between breast cancer and BCL2 C(-938) A gene polymorphism in our study (7.5% vs 31%). Bhushann et al. showed the relationship between this polymorphism and breast cancer in contrast with our study [29].

In our study, no statistically significant relationship was found between XRCC4 G(-1394) T gene polymorphism and breast cancer. In the study of Chiu et al., on the other hand, homozygote or heterozygote expression of –1394 T allele increased the risk of breast cancer. The difference between this study and our study could result from the difference in the incidence of the variant allele [30]. While the incidence of these variant allele as homozygote was 0% in that study, and it was 43% in our study. In their meta-analysis Zhou et al. showed the relationship with this polymorphism and breast cancer [31]. Romanowicz et al. [25] and Saadat M and Saadat S [32] did not find a relationship with this polymorphism and breast cancer in parallel with our study.

Apoptosis and DNA repair are important processes establishing the road leading to cancer development. The proteins utilized during these process play important roles. Gene polymorphisms have key roles in the activity of these proteins. The risks and roles of these polymorphisms and their relationship with clinical parameters have been shown in the literature. Our study has shown that these polymorphisms have significant roles in the development of breast cancer, studying four different gene polymorphisms in the Turkish population for the first time. While we found a relationship only between BAX G(-248) A and XRCC3 Thr241Met between the onset or risk of the disease and the polymorphisms we studied, we found a relationship only between the XRCC3 Thr241Met polymorphism in terms of the severity of the disease.

## Figures and Tables

**Table 1:** Distribution of clinical characteristics of the patients.

Characteristics	Patients
Age (years)	53 ± 12
Range	21–84
Smoking status	
Smoker	61
Nonsmoker	114
Grade	
I+II	99
III+IV	42
Histopathology	
Invasive duct carcinoma	114
Lobular	10
Other	18
Clinical stage	
I+II	98
III+IV	42
Tumor size	
Smaller than 2 cm	66
Larger than 2 cm	76
Estrogen receptor	
Positive	115
Negative	24
Progesterone receptor	
Positive	101
Negative	39
HER-2 receptor	
Positive	30
Negative	112
Triple negative status	
Positive	11
Negative	130
Lymph node status	
Positive	85
Negative	51
Distant metastasis	
Positive	3
Negative	139

^∗^Some of the patient's demographical data could not be found.

**Table 2:** PCR conditions for XRCC3 rs861539, XRCC4 rs6869366, BAX rs4645878, and BCL2 rs2279115 polymorphisms.

Program	Cycle	Time	Temperature (°C)
Initial denaturation	1	4 min	94
Denaturation		30 s	94
Annealing	16	30 s	68–53
Extension		1 min	72
Denaturation		30 s	94
Annealing	30	30 s	55
Extension		1 min	72
Final extension	1	5 min	72
Cooling	Indefinite	Indefinite	4

**Table 3:** PCR and RFLP procedures and expected products of XRCC3 Thr241Met, XRCC4 G(‐1394) T, Bax G(-248) A, and BCL2 C(–938) A genes.

Genes	Primers (forward and reverse)	PCR product	Restriction enzyme	Restriction products
*XRCC3* Thr241Met	5′-GGTCGAGTGACAGTCCAAAC-3′ 5′-TGCAACGGCTGAGGGTCTT-3 ′	456 bp	Nla III (37°C)	Thr/Thr: 316 + 140 bp Met/Met: 211 + 140 + 105 bp
*XRCC4* G(‐1394)T	5′-AGAAGGGCAATCCACCTTTG-3′ 5′-AGCATTAGCGCTTCTCGAG-3′	257 bp	Mbo II (37°C)	GG: 165 + 92 bp TT: 257 bp
*Bax* G(-248)A	5′-CATTAGAGCTGCGATTGGACCG-3′ 5′-GCTCCCTCGGGAGGTTTGGT-3′	109 bp	Msp I (37°C)	GG: 89 + 20 bp AA: 109 bp
*BCL2* C(‐938)A	5′-CTGCCTTCATTTATCCAGCA-3′ 5′-GGCGGCAGATGAATTACAA-3′	262 bp	Bcc I (37°C)	CC: 154 + 108 bp AA: 262 bp

**Table 4:** Distribution of *XRCC3* Thr241Met, *XRCC4* G(‐1394) T, *Bax* G(-248) A, and *BCL2* C(‐938) A genotypes among the controls and patients.

Genotype/allele	Controls, *n*(%)	Patients, *n* (%)	*P* value	OR (95% CI)
Bax G(-248)A				
GG	80 (51)	92 (53)		Reference
GA	74 (47)	58 (33)	0.12	0.68 (0.42–1.10)
AA	4 (3)	25 (14)	*0.002*	5.43 (1.70–15.84)
G allele frequency	0,74	0,69		Reference
A allele frequency	0,36	0,31	0.90	0.92 (0.50–1.72)
BCL2 C(–938)A				
CC	33 (21)	52 (30)		Reference
CA	76 (48)	68 (39)	0.06	0.56 (0.31–1.01)
AA	49 (31)	55 (31)	0.32	0.71 (0.38–1.32)
C allele frequency	0,45	0,49		Reference
A allele frequency	0,55	0,51	0.67	0.85 (0.47–1.54)
XRCC3 Thr241Met				
Thr/Thr (CC)	61 (39)	52 (30)		Reference
Thr/Met (CT)	83 (52)	90 (51)	0.38	1.27 (0.77–2.10)
Met/Met (TT)	14 (9)	33 (19)	*0.009*	2.77 (1.26–6.11)
Thr allele frequency	0,65	0,55		Reference
Met allele frequency	0,35	0,45	0.19	1.51 (0.82–2.79)
XRCC4 G(‐1394)T				
GG	24 (15)	32 (18)		Reference
GT	66 (42)	93 (53)	0.98	1.05 (0.55–2.05)
TT	68 (43)	50 (29)	0.10	0.55 (0.28–1.10)
G allele frequency	0,36	0,45		Reference
T allele frequency	0,64	0,55	0.25	0.68 (0.37–1.26)

## Data Availability

The data is available upon request.
